# Prospective Outcomes of Injury Study 10 Years on (POIS-10): An Observational Cohort Study

**DOI:** 10.3390/mps4020035

**Published:** 2021-05-17

**Authors:** Sarah Derrett, Emma H. Wyeth, Amy Richardson, Gabrielle Davie, Ari Samaranayaka, Rebbecca Lilley, Helen Harcombe

**Affiliations:** 1 Injury Prevention Research Unit, Department of Preventive and Social Medicine, Dunedin School of Medicine, University of Otago, P.O. Box 56, Dunedin 9054, New Zealand; amy.richardson110@gmail.com (A.R.); gabrielle.davie@otago.ac.nz (G.D.); rebbecca.lilley@otago.ac.nz (R.L.); helen.harcombe@otago.ac.nz (H.H.); 2 Ngāi Tahu Māori Health Research Unit, Department of Preventive and Social Medicine, Dunedin School of Medicine, University of Otago, P.O. Box 56, Dunedin 9054, New Zealand; emma.wyeth@otago.ac.nz; 3 Biostatistics Centre, Division of Health Sciences, University of Otago, P.O. Box 56, Dunedin 9054, New Zealand; ari.samaranayaka@otago.ac.nz

**Keywords:** injury, injury outcomes, longitudinal cohort study, Māori health, indigenous health, disability, wellbeing, person-reported outcomes

## Abstract

Injury is a leading cause of disability and is costly. This prospective cohort study extension aims to improve disability, health, and wellbeing outcomes for injured New Zealanders, including for Māori. We will identify predictors and modifiable risk factors of long-term outcomes (positive and negative), and develop an Injury Early Care Tool (INJECT) to inform the implementation of effective interventions to improve outcomes. In the Prospective Outcomes of Injury Study (POIS), 2856 people participated following an injury (occurring between 2007 and 2009) registered with New Zealand’s no-fault accident compensation scheme (ACC). POIS-10 will invite 2121 people (including 358 Māori) who completed a 24-month POIS interview and agreed to follow-up, anticipating 75% participation (*n* = 1591). Interviews will collect sociodemographic characteristics, life events, comorbidities, and new injuries since participants’ 24-month interview, as well as key disability, health, and wellbeing outcomes 12 years post-injury. Injury-related data will be collected from ACC and hospitalisation records 12 years post-injury. Regression models for the main outcomes will examine the direct effects of predictor variables after adjustment for a wide range of confounders. POIS-10 is enhanced by our partnership with ACC, and expert advisors and will benefit injured people, including Māori, through increased understanding of mechanisms and interventions to improve long-term post-injury outcomes.

## 1. Introduction

In New Zealand (NZ), and internationally, injury is a leading cause of disability [1,2]. Furthermore, injury is extremely costly for individuals, families, and society. In the 2018/19 year alone, NZ’s no-fault injury insurer, the Accident Compensation Corporation (ACC), received over 2 million injury claims and spent $4.38 billion supporting injured people [3]. The Prospective Outcomes of Injury Study (POIS) has provided extensive information about a range of disability, health, and wellbeing outcomes experienced by a large representative cohort of injured New Zealanders (*n* = 2856; including 566 Māori), up to 24 months post-injury [4,5]. While a number of participants reported adapting well to life after their injury, many others continued to report significant injury burden two years after the injury had occurred [6,7]. Findings from POIS are consistent with those of a recent systematic review of 29 studies measuring health-related quality of life (HRQL) in general injury populations [8]. Despite significant gains occurring in the first year after an injury, studies found that large functional deficits remained at two years post-injury when compared to population norms and reports of pre-injury health status [8].

### 1.1. Long-Term Outcomes of Injury Internationally

There is accumulating evidence that disability associated with injury is long-term, extending well beyond two years post-injury [9], and that the support needs of injured people may increase over time [10]. In Australia, a longitudinal study of 2757 individuals hospitalised for major trauma found improvements in a number of HRQL domains to 24 months post-injury but detected almost no further improvement in the ensuing 12 months [11]. In fact, overall HRQL was found to decrease over this period as a consequence of pain and discomfort. In a Canadian study, worker compensation claimants with disability onset demonstrated poorer mental [12] and physical health outcomes [12,13] and higher risk of poverty than experienced by the general population at 48 months post-injury [14]. Another study in Australia found high levels of persisting problems 6 years after a serious injury [10], and a prospective cohort study of patients with moderate to severe traumatic brain injury (TBI) in the Netherlands noted a significant decrease in employment out to 10 years post-injury [15]. 

### 1.2. Long-Term Outcomes of Injury in New Zealand

With the exception of findings from our earlier POIS research, little is known about longer-term injury outcomes in NZ. Other NZ studies have described patterns of some specific injury types [16,17,18,19], such as facial fractures, rugby-related injuries, and cycling injuries, but the focus has been on documenting injury incidence [19,20,21] or identifying specific risk factors for injury, e.g., alcohol consumption [22]. Few studies have explored disability, health, or wellbeing outcomes associated with injuries over time. Apart from POIS, two longitudinal studies in NZ have considered such outcomes. One examined individuals with spinal cord injury (SCI; *n* = 118 recruited; 92 followed to 30 months) [23,24], and the other provided important information on the long-term experiences of those who sustained a mild TBI (*n* = 341 recruited; 232 followed to 4 years) [25,26].

### 1.3. Limitations of Existing Research

Both international research and research in NZ have focused on outcomes experienced by people with specific injury types or have been restricted to examining individuals with serious injury only. Consequently, it is not possible to generalise the findings to individuals with different injury types or severities. Furthermore, ‘minor’ injuries (in terms of short-term ‘threat to life’) represent the vast majority of injuries and account for more than two-thirds (67%) of years lived with disability after injury [27,28]. Individual perceptions of an injury may make a more important contribution to post-injury outcomes than objective indicators of injury severity. A Swiss study of 85 injured people followed to 36-months after admission to an intensive care unit found that outcomes (such as paid work) were best predicted by individuals’ own appraisal of their injury severity and ability to cope with the injury rather than by ‘objective’ assessments of injury severity or type [29]. This points to the importance of collecting person-reported data in studies examining outcomes of injury, although many studies have relied solely on routinely collected hospital/emergency department (ED) administrative data. Our NZ POIS research to 24 months post-injury also found considerable post-injury burdens experienced by people with injuries traditionally regarded as being of lesser concern, e.g., people not hospitalised for injury [30,31].

### 1.4. Contribution of POIS

POIS is an internationally unique study that has evaluated outcomes over time among New Zealanders injured between 2007 and 2009, including those hospitalised and non-hospitalised, and with a wide range of injury types (e.g., fractures, sprains and strains, TBI) that occurred in a variety of settings (e.g., road, work, home, sports facilities) and from a range of causes (e.g., motor vehicle crash, assault, work-related) [5]. Participants were randomly selected from one of four geographical regions of NZ using ACC’s entitlement claims register. Individuals on this register have sustained injuries serious enough to warrant compensation for treatment and/or income support. From its inception, POIS was explicitly designed to provide information relevant to Māori [32,33]. Recruitment of participants did not cease until the cohort included 20% Māori, enabling analyses of outcomes (and predictors) specific to Māori participants up to 24 months post-injury [6,34,35,36,37]. We understand POIS to be the largest longitudinal cohort study of injured Māori. This is important as Māori are known to experience marked health inequities compared to non-Māori, including for injury and disability [2,3]. To help address inequities, knowledge about outcomes experienced by Māori and predictors of those outcomes is necessary. 

POIS participants were interviewed 3, 12, and 24 months post-injury, providing information about their pre-injury characteristics (at the 3-month interview) and their injury experiences and outcomes at each follow-up time point [38]. Self-reported interview data were linked with information from large administrative datasets, including claims e-data from ACC (e.g., earnings-related wage compensation, health professional utilisation, treatment costs, and additional injury events) and injury-related hospitalisations recorded in the Ministry of Health (MoH) national minimum data set (NMDS) [39,40]. Results for the whole cohort, and specifically for Māori [6,34,35,36,37], revealed key predictors of disability [30,31,41], participation in paid work [36,42,43,44,45] and unpaid activities [46], other health outcomes including subsequent injury events [40,47], HRQL [46], physical functioning [7], and wellbeing outcomes [33,48] using validated measures. Unlike other longitudinal studies with follow-up rates of 30–50%, POIS achieved a high rate of follow-up at each data collection point, with a 79% follow-up rate at 24 months post-injury. 

Findings from POIS have been of proven value to NZ crown entities, including ACC, resulting in changes in long-term priorities and a renewed focus on life-course perspectives, outcomes, and hauora/wellbeing. POIS has also had significant international impact, contributing data to the six-country Validating and Improving Injury Burden Estimates (Injury-VIBES) Study, which aimed to provide valid estimates of the burden of non-fatal injury by combining data from prospective cohort studies of injury outcomes undertaken in the UK, USA, Australia, NZ, and the Netherlands [49].

### 1.5. Benefits of Additional POIS Follow-Up

POIS-10, by leveraging off our earlier POIS project, will be the first and largest study to quantify the disability, health, and wellbeing outcomes for New Zealanders by conducting in-depth telephone interviews with participants who are now 12 years post-injury. These interview data will be linked with ACC e-data and information on injury-related hospitalisations occurring in the 10 years since the last POIS interview was conducted. Developmental work for this follow-up study found that 1649 (73%) of 2256 POIS participants reported at least one of a range of adverse outcomes (including disability, poor HRQL, and non-return to paid employment) at the 24-month follow-up. Of course, various factors such as comorbidities or labour market dynamics may influence outcomes such as disability and paid employment. However, 1044 (46%) people also specifically reported they had not recovered from their original injury at the 24-month time point. Therefore, it is important to understand New Zealanders post-injury outcomes into the longer term, to identify factors predicting these outcomes, and to inform the development of effective and timely interventions. Given the current lack of information about long-term post-injury outcomes globally, POIS-10 will be of national and international interest and importance, as it will be (to the best of our knowledge) the first prospective study to investigate a range of outcomes to 12 years post-injury, identify factors predicting these long-term outcomes, develop a tool to assist agencies and health professionals to identify individuals at high risk of poor outcomes, and guide prevention-oriented supports targeting key predictors.

### 1.6. POIS-10 Aims and Objectives

POIS-10 aims to contribute to understanding and improving disability, health, and wellbeing outcomes for injured New Zealanders, including specifically for Māori. To achieve this, we will identify modifiable risk factors for adverse long-term outcomes and predictors of positive outcomes and develop a prediction tool to inform the timely implementation of effective interventions to improve outcomes. 

Specifically, POIS-10 will address the following objectives:

1. Describe significant life events, employment, comorbidities, injuries and injury-related hospitalisations experienced by POIS-10 participants over the past 10 years, as people were last interviewed 24 months after the injury, which led to their recruitment to POIS (referred to as the ‘sentinel injury event’ (SIE)), including specifically for Māori;
2. Investigate 12-year SIE outcomes (disability, health, and wellbeing) experienced by all POIS-10 participants and for key subgroups;
3. Determine which characteristics (including baseline sociodemographic and health related, SIE related, and post-SIE related) predict outcomes for POIS-10 participants at 12 years post-SIE;
4. Analyse key outcome trajectories over time;
5. Develop a POIS-10 Injury Early Care (prediction) Tool (INJECT) for long-term outcomes, informed by predictors found to be of importance.

## 2. Experimental Design

**Figure 1:**
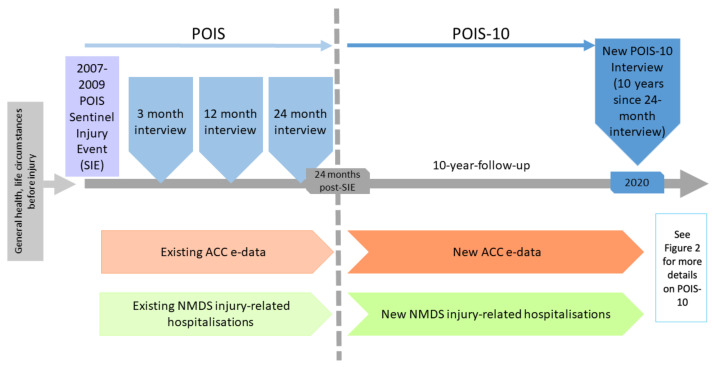
Overview of POIS-10 in relation to the earlier POIS project.

Cohort study extension; following participants to 12 years post-injury [5]. To achieve POIS-10 aims, we will collect detailed interview data from participants during 2020–2021, which we will then link to their administrative e-data from ACC about the SIE, new injuries and service utilisation, and from the MoH’s NMDS about injury-related hospitalisations, to 12 years post-SIE. Figure 1 provides an overview of the study, data collection timepoints, and sources.

## 3. Procedure

### 3.1. Participants

POIS participants (*n* = 2856, including 20% Māori) were aged 18–64 years at the time of the SIE (occurring between 2007 and 2009) and were recruited via ACC’s entitlement claims register (see Figure 1) [5]. Of these, 2256 (79%) completed the 24-month POIS post-injury interview. Eligible POIS-10 participants are a subset of this group who agreed to future follow-up (*n* = 2121, including 358 Māori). Due to our strong POIS follow-up rates, engaged participants, and multiple contact details and methods, we anticipate a POIS-10 participation rate of at least 75% (*n* = ~1591 participants).

### 3.2. POIS-10 Recruitment

During the earlier POIS interviews, we collected several alternative contact details e.g., email addresses and phone numbers for both participants and significant others they identified (e.g., whānau or close friends), to help us contact people for POIS-10 follow-up. For participants we are unable to track using these available contact details, we will: (i) trace using the electoral roll or (ii) trace via updated contact details held by ACC (following ethical approval), as done in the earlier POIS project. We know from our research that 58% of POIS participants went on to experience a new injury event reported to ACC in the 24 months following their SIE [40]. Therefore, with ACC as an active collaborator, we expect to obtain reliable updated contact details for most harder-to-track POIS-10 participants.

### 3.3. Data Collection

As shown in Figure 1, POIS-10 will gather data to 12 years post-SIE via interview and e-data collection during 2020–2021:

1. Interviews to 10 years since last POIS data collection: Interviewer-administered computer-assisted telephone interviews (CATIs) will be conducted by a team of highly trained interviewers. Based on our earlier POIS interviews, each interview is expected to be up to 1 h in duration. Interview questions include a range of sociodemographic characteristics [50,51,52], new major life events [53], and comorbidities [54] occurring over the 10 years since the last POIS data collection (24 months post-SIE); and key disability [55], health [56,57], and wellbeing [58,59,60] outcomes at 12 years post-SIE. Interview questions will, as for POIS, align with the World Health Organization International Classification of Functioning, Disability, and Health framework [61].
2. E-data between 24-month and 10-year follow-up: We will also obtain administrative e-data from ACC about new injuries occurring during the past 10 years (e.g., funded health services, support and claims processes, and earnings-related compensation) and also about ongoing claim entitlements from the original SIE. From the NMDS, we will collect administrative data about any injury-related hospitalisations occurring over the past 10 years.

### 3.4. Types of Data Collected

In addition to the new administrative data from ACC and the NMDS, the POIS-10 interviews will collect information from each of the following categories:

#### 3.4.1. Sociodemographic Characteristics

Information about ethnicity, current living arrangements, and income, 12 years post-SIE, will be asked using standard NZ Census questions [51]. Participants will also be asked to report experiences of racism using questions from the NZ Health Survey 2016–2017 [62].

#### 3.4.2. Major Life Events and Comorbidities over the 10 years since Last Interview

Major life events that have occurred over the last 10-year period will be assessed via the Social Readjustment Rating Scale [53], as in POIS. This questionnaire is designed to evaluate the cumulative impact of a wide range of common stressors (e.g., divorce, death of a family member). Comorbidities will be measured using a list of 21 conditions, previously used by NZ’s MoH and in POIS [41,54]. We will also estimate changes in employment and job turnover in the period between 24 months post-injury and the 10-year follow-up by collecting the number of jobs held and relevant job titles over the last 10 years, and brief reasons for each job change (i.e., health/disability reasons, workplace restructuring, job satisfaction).

### 3.5. Key Outcomes

**Figure 2:**
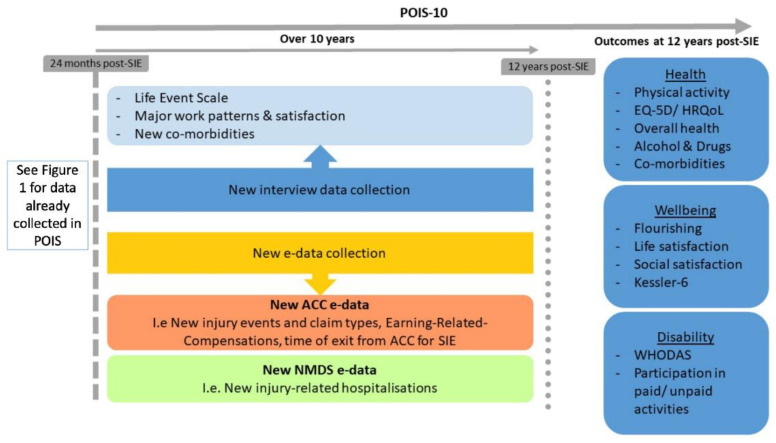
New POIS-10 data collection.

Figure 2 provides an overview of POIS-10 data collection at 12 years post-SIE, including information about three key outcome groups (disability, health, and wellbeing). 

#### 3.5.1. Disability

A comprehensive assessment of disability will occur using two multi-dimensional measures. The 12-item WHODAS (used in POIS) is a brief and widely used questionnaire that measures perceived activity limitations in the past month in relation to six key domains of function: (1) understanding and communication; (2) self-care; (3) mobility; (4) interpersonal relationships; (5) work and household roles; and (6) community participation [55]. Participation in paid work and unpaid activities will also be collected at 12 years post-SIE [46]. Work participation, which was also assessed at 3, 12, and 24 months post-SIE, will be determined using the question: ‘Which of the following best describes your paid work situation now?’ [44] For those in paid work, hours and days worked per week, type of contract, multiple job holding status, job strain and support, job satisfaction, and physical factors associated with work will be collected, as we have done before [45].

#### 3.5.2. Health

HRQoL will be measured using the EQ-5D [56], as in POIS [47,48,63,64]. This assesses health across five key domains of mobility, self-care, usual activities, pain/discomfort, and anxiety/depression. Physical activity will be assessed with a question from the New Zealand Physical Activity Questionnaire (short-form) [65], asking respondents to recall the number of days they engaged in physical activity during the previous week. Alcohol use will be captured by the AUDIT-C [57], comprising three questions designed to identify alcohol use disorders. We will also collect data on additional substance use as in POIS interviews [66]. Additionally, a single item will ask participants to rate their overall health status [67]. As mentioned, complementing self-reported health outcomes, injury claim data and service utilisation from ACC’s e-data, and injury-related hospitalisations during the 10-year follow-up period will be identified within the NMDS-adding to the individual-level information we have already collected to 24 months post-SIE in POIS. 

#### 3.5.3. Wellbeing

Depression and anxiety symptoms will be measured using the 6-item Kessler Psychological Distress Scale [59], a brief instrument for the detection of serious mental illness. Life satisfaction and social satisfaction will be assessed as in previous POIS interviews [31,58]. Finally, a brief measure of flourishing (understood as social–psychological prosperity), the 8-item Flourishing Scale [60], will be administered. This questionnaire has been validated in a large sample of New Zealanders, including 1232 Māori [68]. Our data collection has been deliberately designed to inform analyses of both positive and adverse outcomes, and the Flourishing Scale is important in this regard.

### 3.6. Ethical Approval and Data

POIS-10 has received ethical approval from the Health and Disability Ethics Committees New Zealand (MEC/07/07/093/AM07). All participants will be asked to provide consent for interview and data collection from ACC or the NMDS. Participant identifiers will be held securely on password-protected computers and/or locked offices, which are only accessible to the study team members. Electronic data from ACC/MoH will be stored according to agency protocols. All data will be de-identified prior to analysis, and no potentially identifiable person-level findings will be reported.

## 4. Expected Result

In partnership with ACC and a team of expert advisors (including two people with lived experience of injury), POIS-10 will quantify the long-term disability, health, and wellbeing consequences of a broad range of injuries for the first time in NZ, including specifically for Māori. POIS-10 will collect detailed person-level data; findings will therefore result in knowledge beyond the reach of administrative datasets alone (e.g., those of ACC, or NZ’s Integrated Data Infrastructure). The likelihood of POIS-10 achieving high impact is illustrated by our earlier POIS project, which informed ACC’s research strategy, focus on outcomes, and provided knowledge previously unavailable to ACC and others about person-level disability [6,30,31], health [7,48,69], and wellbeing outcomes [33,42,43,44,45]. Importantly, POIS-10 will provide knowledge about outcomes specifically for Māori [32]. Analyses of data provided by Māori will be led by Dr Wyeth (co-principal investigator; Kāi Tahu), along with other Māori advisors involved in POIS-10. 

### 4.1. Analyses

To address Objectives 1 and 2, summary variables of interest will be derived from the linked dataset. We will estimate (with 95% confidence intervals) prevalence, incidence, and changes over time (trends), both overall (~*n* = 1591) and for Māori specifically (~*n* = 269). These steps are crucial in understanding the data in preparation for statistical model building. For Objective 3, we will first combine POIS data with data obtained in POIS-10 then develop regression models (generalised linear models for continuous outcomes, modified Poisson regression with robust standard errors for binary outcomes) [70] for each of the main outcomes at 12 years post-SIE to examine the direct effects of the postulated predictors after adjustment for a wide range of confounders. Decisions around the inclusion (or not) of predictors and potential confounders will be informed by existing literature, previous POIS analyses to 24 months post-SIE, and findings from Objectives 1 and 2. We will also compare 12-year post-sentinel injury outcomes between sub-groups of POIS-10 participants; e.g., comparison of 12-year outcomes between the 46% who reported non-recovery at 24 months, with the 54% reporting recovery, will be of particular interest, as will the long-term comparison between the 25% hospitalised post-SIE and the 75% not hospitalised. Additionally, ACC e-data will be used to identify when participants exit ACC’s scheme for their SIE; outcomes will be compared between those who exited early, exited later, or have not yet exited. Given the large sample followed to 24 months, even if POIS-10’s response rate is lower than the 75% anticipated (e.g., 65%), we will still have sufficient statistical power to meet our objectives for the cohort overall. We will conduct similar analyses explicitly for POIS-10 Māori participants. While statistical modelling for Māori outcomes will have less power, it is likely to be sufficient to show a level of consistency or inconsistency with overall population findings. Assuming 10% have a particular binary outcome, our likely POIS-10 Māori cohort (~*n* = 269) will be sufficient to estimate around 3 parameters in a single model. More parameters than this will be possible when the outcome is continuous. Objective 4 is focused on outcomes at multiple time points to 12 years post-SIE. Quantitative analyses will first describe key outcome trajectories over time (i.e., recovery pathways), as described previously [7]. Models will be extended to cope with repeated measurements (i.e., generalised estimating equation models, generalised linear mixed models) [41]. Exposure-by-time interactions will be included, as appropriate, for repeated measures. Although our track record of follow-up and fully completed interviews is strong, any loss to longer-term follow-up is highly unlikely to be random. We will consider multiple imputations or other techniques such as Inverse Probability Weighting to address missingness and conduct sensitivity analyses as appropriate [71,72]. We will conduct similar analyses specifically for POIS-10 Māori participants. Objective 5 explicitly recognises the value of prediction modelling [73]. Following an approach used with a smaller cohort of Australian compensation claimants to develop prediction models of disability at 72 months post-injury, we will develop an early care prediction tool for NZ, identifying those who will most benefit from targeted additional early support after injury to reduce ongoing personal burdens and societal costs [10]. Each predictor in a multivariable regression model will be assigned a number of points (index directly corresponding to the size of the predictor’s model-based coefficient). Points will be summed across predictors for each participant to produce an overall individual-level score using the developed POIS-10 Injury Early Care Tool (POIS-INJECT).

### 4.2. Dissemination

Lay summaries of key results from POIS-10 will be shared with study participants by post and/or email and will also contain the web address for the study blog where peer-reviewed publications will be listed as they become available. Results will also be disseminated by, and discussed with, Māori providers and organisations in presentations and written reports. Results will be published in national and international peer-reviewed journals and presented at conferences. Dissemination meetings, nearer to the close of POIS-10, will focus on the POIS-INJECT tool.

## Data Availability

Not applicable.
